# Efficacy of rituximab in non-paraneoplastic autoimmune retinopathy

**DOI:** 10.1186/s13023-017-0680-7

**Published:** 2017-07-15

**Authors:** Katherine Boudreault, Sally Justus, Jesse D. Sengillo, Kaspar Schuerch, Winston Lee, Thiago Cabral, Stephen H. Tsang

**Affiliations:** 1 Department of Ophthalmology, University of Montreal, Montreal, Canada; 2 Jonas Children’s Vision Care, and Bernard & Shirlee Brown Glaucoma Laboratory, Columbia University Medical Center, New York, NY USA; 3 Department of Ophthalmology, Columbia University, New York, NY USA; 4 State University of New York Downstate Medical Center, Brooklyn, NY USA; 5 Department of Ophthalmology, Federal University of Sao Paulo (UNIFESP), São Paulo, Brazil; 6 Department of Ophthalmology, Federal University of Espírito Santo (UFES), Vitória, Brazil; 7 Department of Pathology & Cell Biology, Institute of Human Nutrition, College of Physicians and Surgeons, Columbia University, New York, NY USA

**Keywords:** Autoimmune retinopathy, Rituximab, Treatment, Multi-modal imaging, Electroretinography

## Abstract

**Background:**

Autoimmune retinopathy (AIR) is a rare but potentially blinding condition that is often underdiagnosed. Common features in AIR presentation include rapidly progressive vision loss with abnormal electrophysiological responses of the retina associated with positive anti-retinal antibodies. AIR is also challenging to treat, and thus, the introduction of new potential therapeutic agents is welcomed. The goal of this communication is to assess the effects of rituximab infusions on electroretinogram (ERG) responses and visual function outcomes in patients with non-paraneoplastic autoimmune retinopathy (npAIR).

**Results:**

Following infusion(s), three out of five patients showed no evidence of disease progression or improved, while two patients continued to progress on ERG. One patient demonstrated improvement in visual acuity (2 lines) in both eyes. ERG responses provided objective monitoring of patients’ visual function and response to immunosuppression over time.

**Conclusions:**

These findings suggest that patients with npAIR unresponsive to other immunosuppression therapies may benefit from rituximab infusion, although stabilization rather than improvement was more frequently the outcome in our case series. Furthermore, regularly scheduled ERG follow-up examinations are recommended for monitoring patients’ progression during treatment.

## Background

Autoimmune retinopathies (AIR) comprise a spectrum of relatively uncommon autoimmune retinal diseases. Although AIR have been studied for the past 40 years [1, 2], they remain difficult to diagnose [3] and treat. AIR include such conditions as paraneoplastic autoimmune retinopathy (pAIR), which can be further subdivided into cancer-associated retinopathy (CAR) and melanoma-associated retinopathy (MAR). In the absence of malignancy, the condition is referred to as non-paraneoplastic autoimmune retinopathy (npAIR). A commonality uniting pAIR and npAIR is that in both conditions, the integrity and function of various retinal cells, including cones, rods, and bipolar cells, are affected by antiretinal antibodies (ARAs) that are believed to arise from molecular mimicry [4]. The cell types that are most affected in each patient, and thus the initial signs and symptoms, likely depend on which retinal proteins are targeted by the ARAs [5–7]. Consequently, this causes heterogeneity in clinical presentation among patients, including central vision loss, variable changes in visual field, retinal structure, and morphology [8]. Recently, a panel of experts proposed a list of key diagnostic criteria for AIR, among which included: the absence of an apparent cause for visual dysfunction, an abnormal ERG, and the presence of serum ARAs [9]. Until now, there is no standard therapy or established treatment protocol, and patient outcomes following intervention are variable. However, a drug called rituximab has garnered interest as a potential treatment option.

Rituximab is a monoclonal antibody that binds to CD20, a non-glycosylated protein expressed on the surface of B lymphocytes (B-cells), inducing B-cell lysis [10]. It was first approved by the FDA for the treatment of B-cell lymphoma, although recently it has been applied to a variety of autoimmune disorders [11]. However, its use has not been extensively explored for immune-related retinal conditions, and only case reports and one case series have discussed rituximab administration for patients with AIR [12–19]. Here, we present a case series of five patients exploring the effects of rituximab therapy for the treatment of npAIR as assessed by electrodiagnostic testing. Our aim is to provide a reference for clinicians who are seeking new options for managing this complicated disease and to demonstrate the utility of the ERG as a means of assessing response to immunosuppression in npAIR.

## Methods

We performed a retrospective review of all cases of npAIR diagnosed at the Edward S Harkness Eye Institute at New York-Presbyterian Hospital (NYPH) between 2009 and 2016. Five cases were selected based on the following inclusion criteria: (1) they received at least one rituximab infusion during their disease course, and (2) they had a minimum of a six-month follow-up to assess visual function with electrodiagnostic testing, visual acuity, and multimodal imaging, as well as visual field testing when available. The diagnosis of npAIR was based on peer-reviewed diagnostic criteria [9].

Detection of ARAs in all patients was confirmed by one of two laboratories: The Ocular Immunology Laboratory located at Oregon Health & Science University (Portland, Oregon) or The University of California at Davis Laboratory (Davis, California). Autoantibody detection was performed as previously described [20, 21]. Briefly, serum was collected from patients and the presence of anti-retinal antibodies was determined by western blot analysis. Western blot band thickness was compared between tests and used to assess the change in response for specific antibodies over time.

Full-field electroretinograms (ffERGs) (Diagnosys LLC, Lowell, Massachusetts, USA) were recorded from both eyes with DTL electrodes according to the standards from the International Society for Clinical Electrophysiology of Vision (ISCEV) [22] in both scotopic and photopic states. When 30 Hz flicker amplitudes were lower than 5 microvolts (μV), Burian-Allen contact lens electrodes were used to record the electric responses. The amplitudes and implicit times obtained from both eyes of each patient were compared with age-matched normal values, in which the boundary of normal limits represented two standard deviations from the mean.

The main outcome was the effect of rituximab on visual function before and after treatment and was determined based on two independent criteria: (1) stability or improvement in ERG scotopic and/or photopic response, using the last response before treatment with rituximab as the baseline (ratio = 1); and (2) improvement or stability in best corrected visual acuity (BCVA), using the last visual acuity measurement before initiation of treatment with rituximab as the baseline (ratio = 1). Ratios were calculated for ERG and BCVA by comparing the post-treatment response to baseline response ($\frac{after\  rituximab}{just\  before\  rituximab}$), such that values greater than 1 indicate improvements in visual function, and values less than one indicate declines in function. Secondary outcomes included subjective stability or improvement on visual field testing; retinal structure, as assessed by spectral-domain optical coherence tomography (SD-OCT) when available; and assessment of antibody titer after rituximab infusions. The following data were retrieved retrospectively for each patient: demographics (age, gender); medical history (history of other autoimmune diseases); clinical features; non-rituximab treatment data (treatment(s), response); and rituximab treatment data (dose, adverse reactions, response).

## Results

### Demographics

**Fig. 1:**
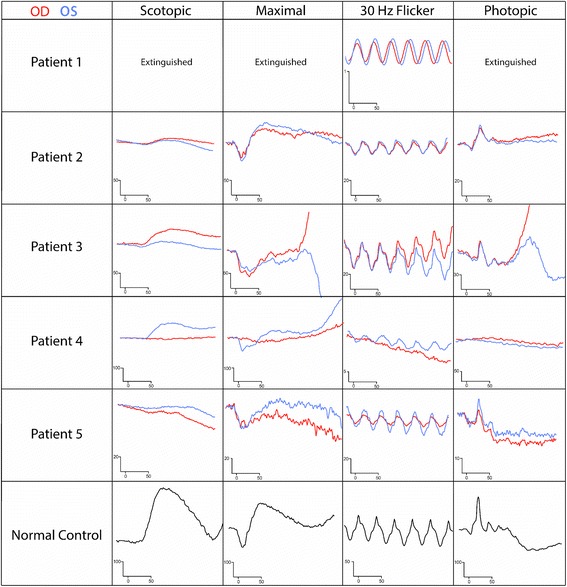
Scotopic and photopic ERG curves at presentation for each patient. Patient 1 (P1) ERG values are presented in the top row, and each patient’s data sequentially follows, with a normal control provided on the bottom row. Scales vary between patients. OD shown in *red*, OS in *blue*

In the present study, patients ranged between 10 and 70 years of age; one was an African-American male, and four patients were Caucasian females. Two patients were originally diagnosed with suspected inherited retinal dystrophies, but in both cases, visual acuity and/or visual field loss deteriorated rapidly, which is not consistent with the typically slow natural history of inherited retinal dystrophies. The median follow-up period was 51 months. Three patients (P1, 2, and 5) had a past medical history of systemic autoimmune disorder, and P4 was diagnosed with Crohn disease 2 years after the initial npAIR diagnosis (Table 1). Four were on other immunosuppressants prior to rituximab, with an initially positive response to treatment in three patients that eventually failed, prompting initiation of rituximab. Four patients presented with rod-cone dysfunction, and one patient (P4) presented with cone-rod dysfunction on electroretinography (Fig. 1).

**Table 1:** Clinical characteristics of npAIR patients

Case#/Sex/Age (y)	Symptoms onset before confirmed dx (m)	Other autoimmune disorder(s)	Total Follow-up duration (y,m)	WB bands at presentation (kDa)	npAIR/RP	BCVA OS/OD at presentation	Immunosuppressors before rituximab/Response before failure
1/F/61	24	Psoriasis, HypoTSH	6y9m	33, 45, 55, 64, 72, 90	Yes/No	20/150 20/150	MMF, I/Improved and stabilized × 60 months on R
2/M/65	28	Cutaneous LE	4y3m	40, 46 (enolase), 68	Yes/Yes	20/40 20/40	MMF/No response after 7-month trial
3/F/16	36	None	2y10m	23 (HSP27), 28, 34, 36, 39, 46 (enolase), 62	Yes/Yes	20/40 20/40	None
4/F/10	12	Crohn Disease	2y2m	28, 92	Yes/No	20/150 20/125	C, P, MMF, IVIG/Improved × 5 months
5/F/70	6	Hashimoto Thyroiditis, Sjogren syndrome	4y6m	42	Yes/No	20/25 20/30	MMF, P/Initial improvement, than compliance variable with stepwise decrease in visual function

*Y* years, *m* months, *kDa* kilodalton, *WB* western blot, *BCVA* best corrected visual acuity, *HypoTSH* hypothyroidism, *Cutaneous LE* Cutaneous Lupus Erythematosus, *MMF* mycophenolate mofetil, *I* infliximab, *R* rituximab, *C* cyclosporine, *P* prednisone, *IVIG* intravenous immunoglobulins

### Response to rituximab across patients

**Fig. 2:**
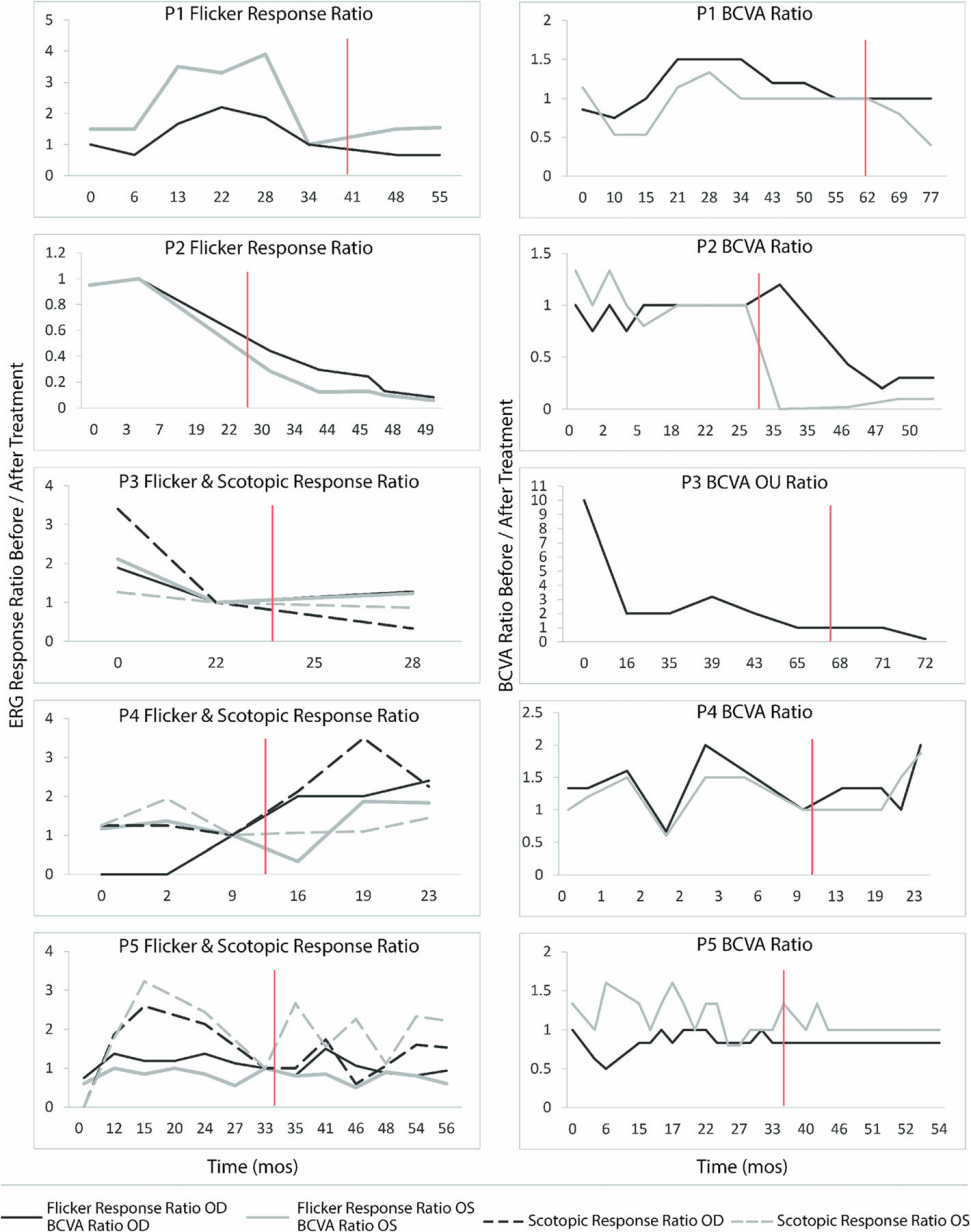
ERG/BCVA ratio variation with time. ERG (*left*) and BCVA (*right*) ratio comparing response from last appointment before rituximab administration to each subsequent (or previous) appointment (After/Before). Data from P1 is depicted in the top, and each patient’s data sequentially follows. *Red line* signifies time point of first rituximab administration

Median follow up period after first rituximab infusion was 15 months (Table 2). The rituximab treatment regiment varied between patients with regards to the number of infusions, dosage, and interval between doses, which was adjusted based on patients’ B cell count and specialist preferences. After rituximab infusions, patients 1 and 5’s flicker ratio stabilized and P4’s improved, while patients’ 2 and 3 flicker and/or rod ratios showed a rapid decline below a ratio of 1. The best-corrected visual acuity ratio correlated closely with ERG flicker ratio in most cases, except for P1, who developed a cataract in one eye. Only one patient (P4) demonstrated improvement in visual acuity (2 lines with Snellen) in both eyes (Fig. 2). One patient (P5) developed more frequent sinus infections, nodular scleritis, and zoster ophthalmicus during the course of her treatment. Table 3 summarizes patient outcomes.

**Table 2:** Rituximab treatment details and responses

Case	Indication	Rtx induction dosage	Rtx maintenance dosage	ERG response	Visual field after Rtx	OCT of the macula	Visual acuity	Atb results before Rtx	Atb results after Rtx/time when sample was collected	Outcome after Rtx	Follow up after Rtx (m)	Rtx stopped because of side effects
1	Failure on other tx after initial response	1000 mg X1	1000 mg, 2 month intervals, (total 11 doses)	30 Hz flicker stable	Stability	Improvement in macular edema at 14 months	Stable	45, 50, 56	Absence/(14 months on Rtx)	Stable	14	No
2	No response to other tx	375 mg/m^2^ (750 mg), 4 doses at one week intervals	Same regiment 6 months later	Deterioration	Decreased sensitivity	Progression, increased granularity OD, CRAO OS	Stable	36, 40, 46 (enolase), 68	46 (enolase) (level unknown)/10 months on Rtx	No response	15	No
3	No previous tx	675 mg/m^2^ (1000 mg), 2 doses at one month intervals	-	Deterioration	Not performed	No change	Decreased	23 (HSP27), 28, 34, 36, 39, 46 (enolase), 62	23 (HSP27), 28, 34, 36, 39, 46 (enolase), 62 (same level than before)/5 weeks on Rtx	No response	6	No
4	Failure on other tx after initial response	750 mg/m^2^ (1000 mg), 2 doses at 1 week intervals	Same regiment 13 months later	Every response improved except flicker	Improved	Increased granularity OD, unchanged OS	Improved 1 line OU	Absent after first trial of immuno-therapy	-	Improved	17	No
5	Failure on other tx	375 mg/m^2^ (635 mg), 4 doses at one week intervals	Same regiment 12 months later	Response to rechallenge	Slightly improved	Unchanged, no macular edema	Unchanged	42,45,62, 74	42,45,74 (same level than before)/5 months on Rtx	Stable	21	Yes

*Rtx* rituximab, *tx* treatment, *Atb* antibody, *m* months, *CRAO* central retinal artery occlusion

**Table 3:** Synopsis of patient outcomes

	P1	P2	P3	P4	P5
BCVA	S	S	D	I	S
ERG	S	D	D	I	S
Visual fields	S	D	NA	I	I
ARA (# of bands)	3 → 0	3 → 1	7 → 7	0 → 0	4 → 3
AE	-	-	-	-	2
Overall	Stable	Poor	Unresponsive	Positive	Stable

*S* Stable, *I* Improved, *D* Deteriorated, *NA* not applicable, *AE* Adverse Events

### Cases

#### Patient 1 (P1)

**Fig. 3:**
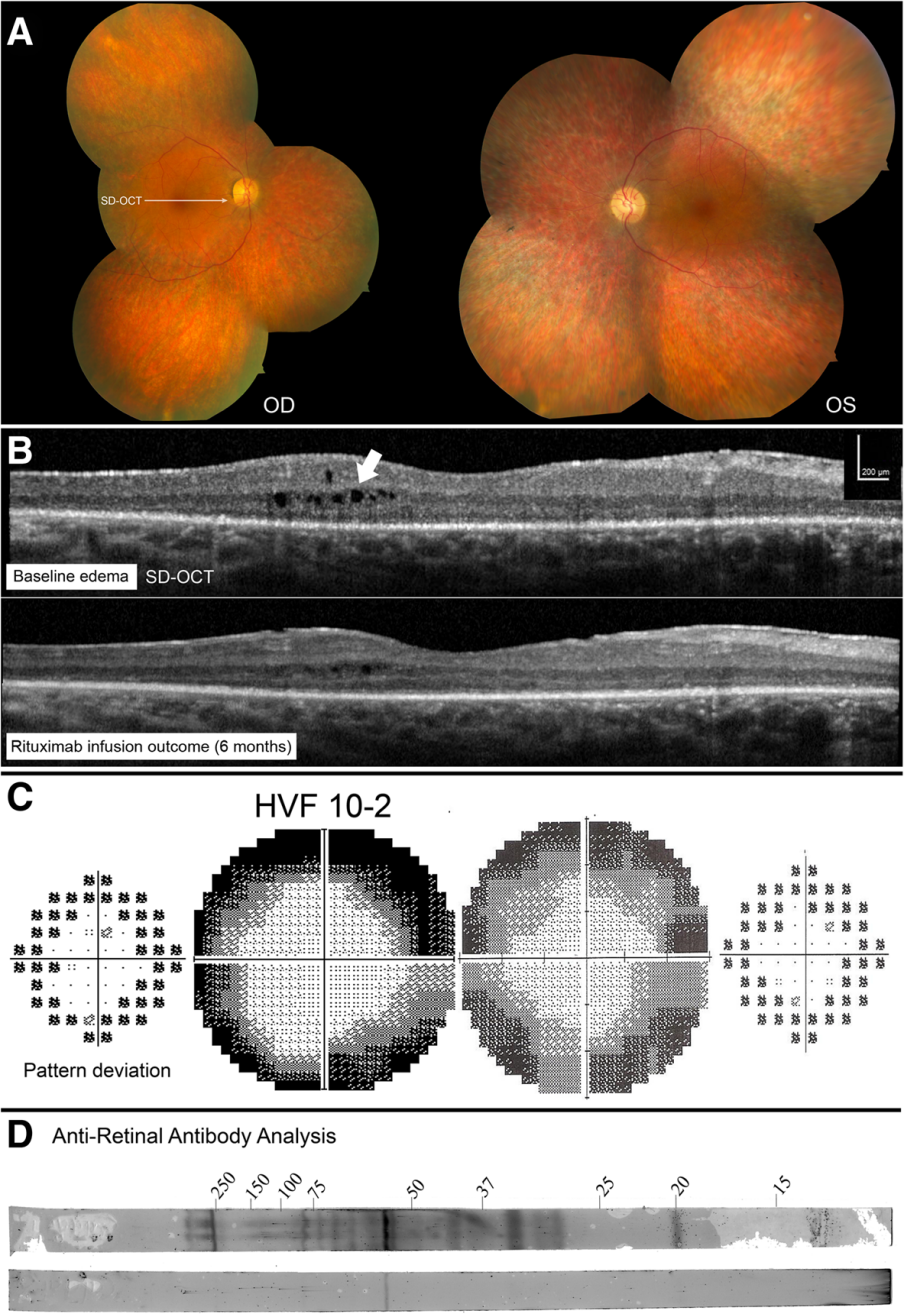
P1 imaging and functional assessments. P1 fundus picture OU at presentation (**a**). OCT line OD showed new cystic macular edema, which improved after rituximab infusions (**b**). Humphrey visual field (HVF) 10–2 OD *grey scale* and pattern standard deviation showed diffuse peripheral loss while on infliximab (2012, *left*), which stabilized after a few months on rituximab infusions (2015, *right*) (**c**). Western blot analysis after 7 months (*top*) and 14 months (*bottom*) on rituximab showed almost complete absence of reaction at follow-up (**d**)

A 61 year-old woman complained of rapid, progressive vision loss. On September 28th, 2009, her vision was recorded at 20/150 bilaterally. The fundoscopic examination revealed extensive mottling of the retinal pigment epithelium (RPE) and limited pigment migration only in the left eye (Fig. 3a). Immunoblot analysis showed reactivity against 33, 45, 55, 64, 72, and 90-kDa proteins, while work-up for neoplastic and infectious causes was negative. Mycophenolate mofetil was initiated, than replaced by infliximab at a dose of 400 mg once every 2 months. At the end of 2011, the first ffERG performed showed an extinguished rod response, and 30 Hz flicker response was approximately 0.3 μV bilaterally (Fig. 1a). Her vision progressively improved to 20/25 in the right eye and 20/30 in the left eye. The ERG showed progressive improvement on 30 Hz flicker response (Fig. 2a). However, around September 2014, her ERG responses deteriorated, she developed macular edema bilaterally, and her vision dropped. Immunoblot analysis showed reactivity against 45, 50, and 56-kDa proteins. Infliximab was discontinued and rituximab infusions were initiated. Repeat immunoblot analysis showed no change 7 months after rituximab institution. In June 2016, her vision had slightly decreased in the left eye, but this was attributed to a posterior subcapsular cataract. Macular edema was slightly improved bilaterally when compared to SD-OCT images taken prior to initiation of rituximab (Fig. 3b). Her visual field and ERG responses have remained within the same range since (Fig. 3c). Fourteen months after institution of rituximab, repeat western blot analysis showed undetectable antibody levels (Fig. 3d). For these reasons, she was declared stable on rituximab.

#### Patient 2 (P2)

**Fig. 4:**
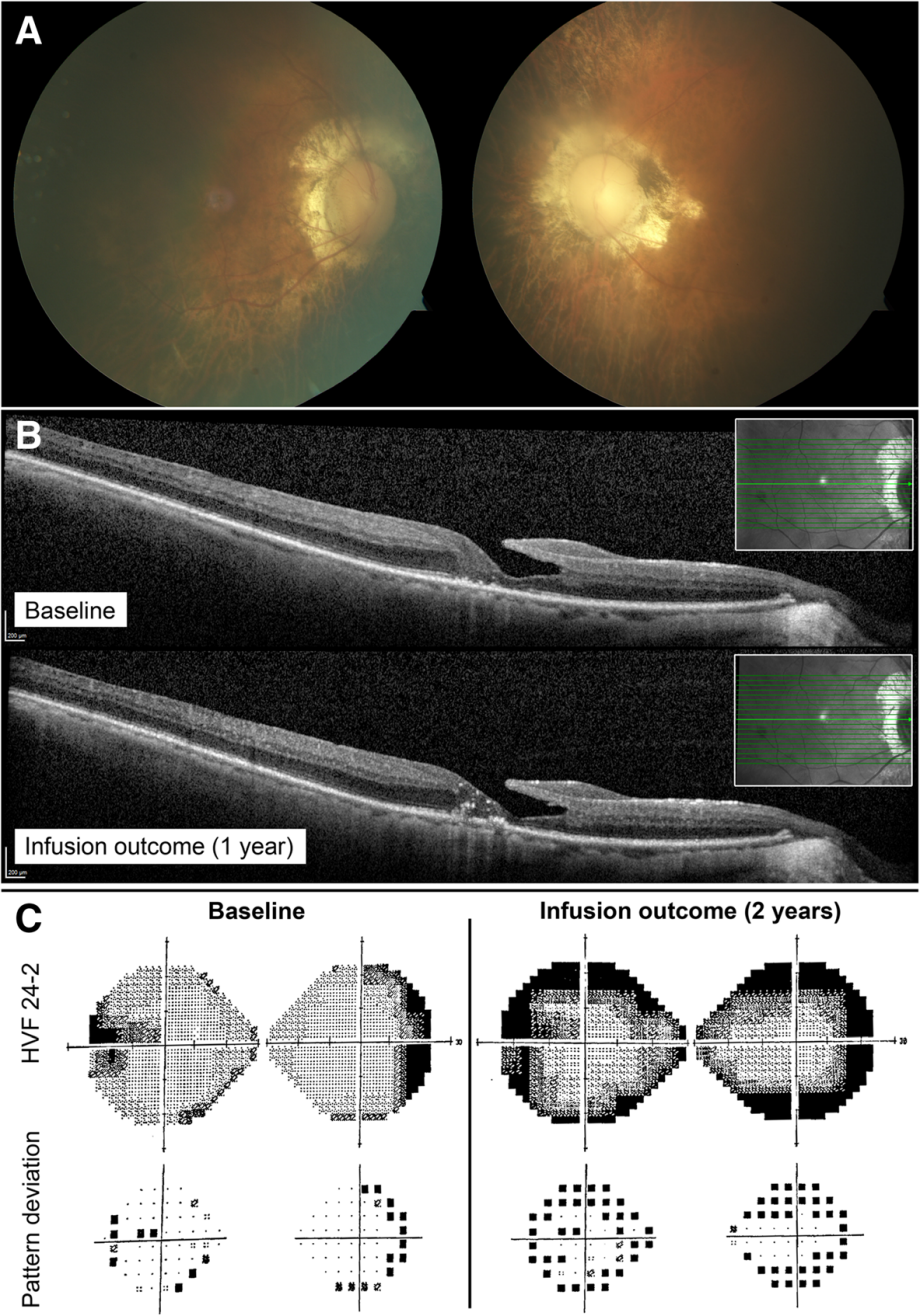
P2 imaging and functional assessments. P2 fundus pictures OU (**a**). OCT line OD showing lamellar hole with cysts in the right eye before starting rituximab (2014, *top*), which continued to deteriorate on rituximab treatment (2015, *bottom*) (**b**). HVF 24–2 OU grey scale and pattern deviation before (2013, *left*) and after (2015, *right*) initiation of rituximab (**c**)

A 65 year-old man reported progressive peripheral vision loss. Initial visual acuity was 20/25 bilaterally. At his first visit in April 2012, his visual acuity dropped to 20/40 bilaterally. Fundus imaging showed myopic fundi, attenuated vessels and mottling of the retina (Fig. 4a). OCT showed a lamellar hole with cysts in the right eye. ffERG showed severe rod-cone dysfunction (Fig. 1b). He was diagnosed with late-onset retinitis pigmentosa. However, in 2013, there was a significantly decreased response on visual field testing. ARA testing was positive for anti-40 kDa, anti-46 kDa (enolase), and anti-68 kDa proteins. Work-up for malignancy was negative, and ERG responses continued to deteriorate (Fig. 2b). The patient was prescribed mycophenolate mofetil 1000 mg twice a day, but seven months later, visual fields were worse and antibody testing, unchanged. The decision was made to escalate treatment and start rituximab infusions. Six months later, OCT of the macula showed a novel granular appearance at the level of the outer retinal layers (Fig. 4b), and the visual field showed continued deterioration in both eyes (Fig. 4c). The patient eventually developed left central vein occlusion and vision dropped to light perception in the left eye. Antibody testing showed reactivity against 46-kDa proteins, and ERG testing comparing responses before starting immunosuppression showed tremendous progression (Fig. 2b). He eventually stabilized with plasmapheresis treatment. Thus, this patient was classified as having a poor outcome on rituximab.

#### Patient 3 (P3)

**Fig. 5:**
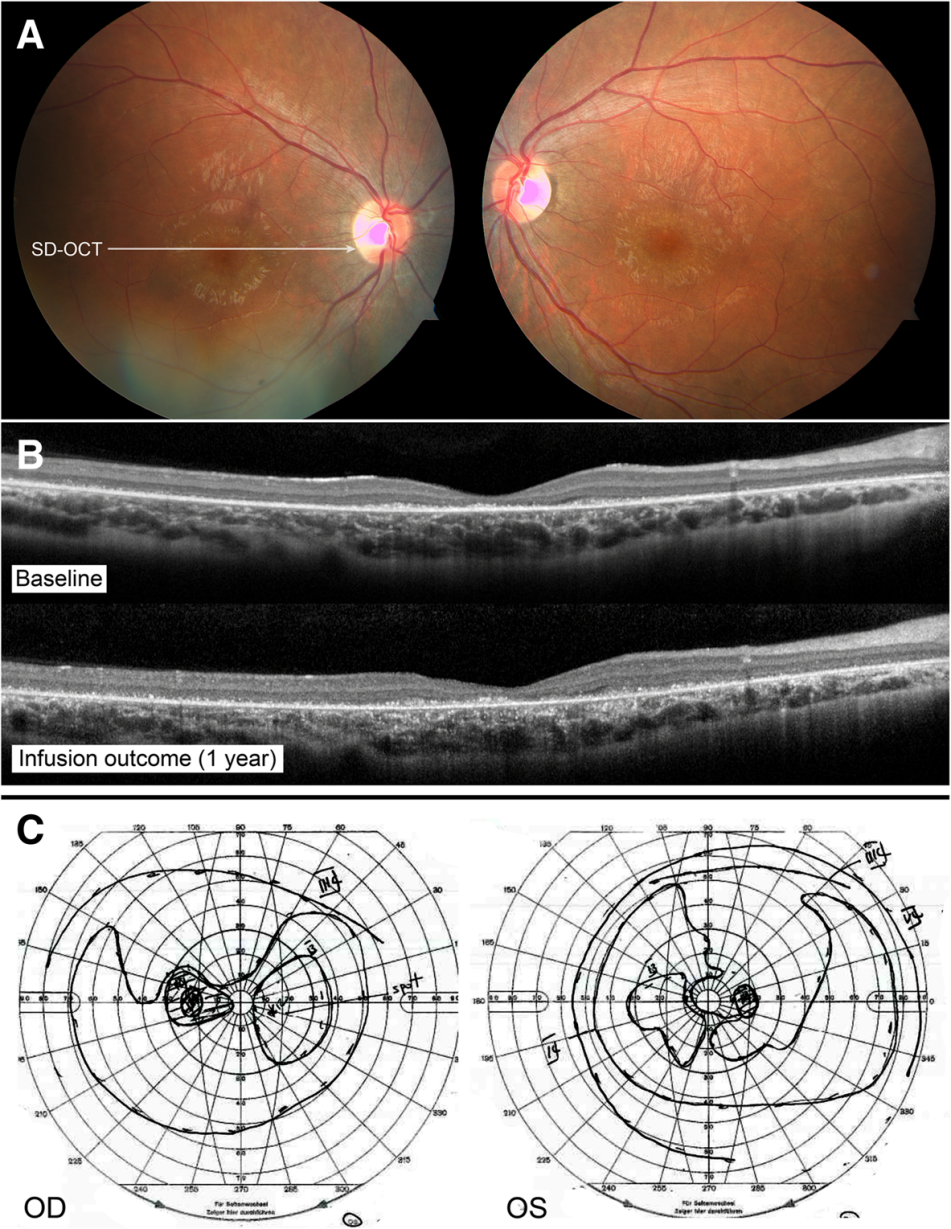
P3 imaging and functional assessments. P3 fundus pictures OU (**a**). OCT line in OD showing retinal thinning and progression of granular deposits at the EZ line between spring 2015 and end of 2015, after rituximab infusion (*top* and *bottom*, respectively) (**b**). Goldmann visual fields in both eyes at first visit (**c**)

A 16 year-old girl without significant past medical history complained of bilateral rapid central visual loss associated with photopsia over the summer of 2010. There was no family history of autoimmune disorders or hereditary retinopathies. The visual loss deteriorated in a stepwise fashion, starting at 20/40 in 2010, to 20/200 in 2011, and eventually to 20/400 bilaterally in 2015. The first dilated fundus examination at NYPH showed a bull’s eye maculopathy (Fig. 5a) and thinning of the retinae with photoreceptor layer loss on OCT, mostly centrally. Goldmann visual fields demonstrated decreased sensitivity, particularly in the maculae in both eyes (Fig. 5c). ffERG showed decreased rod response more so than was observed for cones, with an electronegative appearance (Fig. 1c). In June 2015, testing for ARAs showed reactivity against 23 (HSP27), 28, 34, 36, 39, 46 (anti-enolase), and 62-kDa proteins. Malignancy work up was unremarkable. In the meantime, whole exome sequencing results returned positive for two deleterious mutations in the *MSFD8* gene. Mutations in this gene usually cause neuronal ceroid lipofuscinose (NCL), a neurologic disorder that usually presents with early-onset epilepsy, retinal degeneration, and progressive mental and motor deterioration [23]. In the absence of systemic findings and the rarity of case reports of non-syndromic *MSFD8* patients with late-onset visual loss [24], it was entertained that the patient may have been genetically susceptible to developing vision loss. The combination of a genetic background predisposed towards retinal degeneration as well as numerous anti-retinal antibodies and their proven pathogenicity (enolase and SHP27, especially) could have induced the rapid vision deterioration observed in this patient. In August 2015, rituximab infusions were initiated. Repeated antibody testing five weeks after the first infusion was unchanged. Four months after the second infusion, there was stability in 30 Hz flicker response, but the rod response continued to progress on ERG testing. The vision deteriorated, and an OCT showed worsening granularity when comparing images before and after treatment (Fig. 5b). Thus, this patient was classified as non-responsive to rituximab.

#### Patient 4 (P4)

**Fig. 6:**
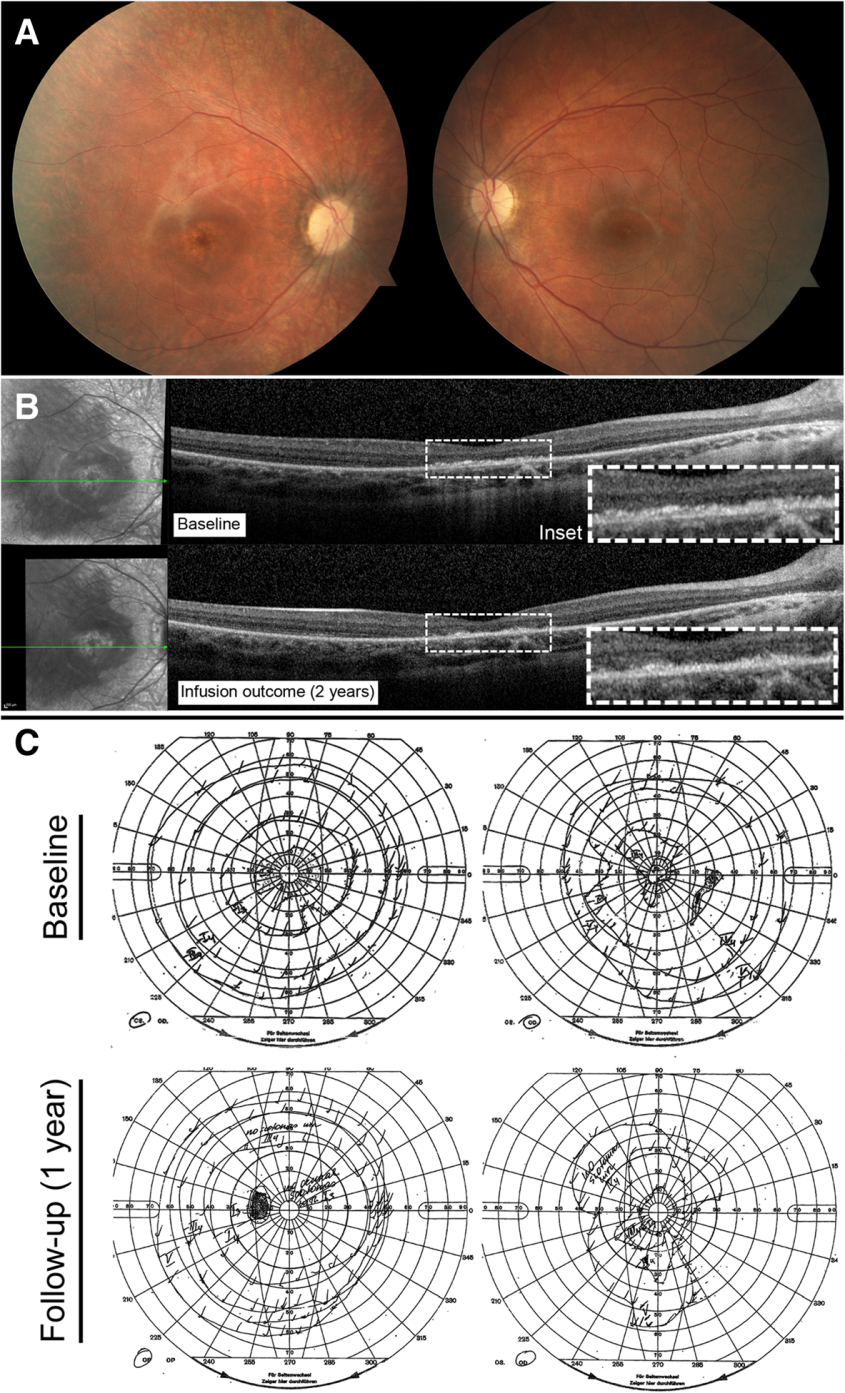
P4 imaging and functional assessments. P4 fundus pictures OU (**a**). OCT line OD showing loss of EZ line centrally and granular deposition before starting rituximab (2014, *top*), which changed minimally on rituximab treatment (2016, *bottom*) (**b**). Goldmann visual field in both eyes before (2014, *top*) and after (2015, *bottom*) initiation of rituximab (**c**)

A 10 year-old girl without significant past medical history and normal ocular exams before symptom onset complained of decreased color vision with phosphene six months prior to visiting the Columbia Eye Institute. In June 2014, BCVA was recorded at 20/150 OD and 20/125 OS. Fundus examination revealed marked vascular attenuation and a large zone of macular RPE and choriocapillaris atrophy in the right eye. The left fundus showed only subtle chorioretinal atrophy centrally (Fig. 6a). OCT demonstrated thinning of the retina and loss of the ellipsoid zone (EZ) line centrally in the right eye as well as partial loss of the EZ line in the left eye. Goldmann visual field showed no response to stimuli smaller than II2 in the right eye and no response to stimuli smaller than I3 in the left eye. ffERG demonstrated asymmetry between both eyes, with a profound decrease in cone response bilaterally and residual rod response in the left eye (Fig. 1d). Full body work-up, serologies for infectious diseases, and genetic testing (retinal panel) returned negative. In June 2014, ARAs showed reactivity against 28-kDa and 92-kDa proteins. Vision further decreased to 20/300 OD and 20/250 OS, and the Goldmann visual field deteriorated. An immunologist initiated intravenous immunoglobulins (IVIg) 2 g/kg with one dose of methylprednisolone at 1000 mg IV, followed by maintenance treatment with prednisone 60 mg, mycophenolate mofetil 1000 mg twice daily, and cyclosporine 150 mg daily. A month later, vision returned to 20/100 bilaterally, and antibody testing came back negative, so a second dose of IVIg was given. However, in January 2015, the visual field worsened again, and ERG responses dropped. Medication was discontinued and the patient received one dose of rituximab. Seven months later, all ERG responses showed improvement or stability except for 30 Hz flicker in the left eye, which declined slightly. Visual acuity improved in the right eye, while an OCT before and after rituximab infusion showed mildly increased granular appearance in the right eye and no change in the left (Fig. 6b). I4 stimulus was now seen centrally in the right eye, and the central scotoma disappeared in the left eye (Fig 6c). Her B-cell level dropped and remained within the appropriate range for 12 months, and she therefore received a second dose of rituximab 13 months later. Subsequently, there was a new response to I2 stimulus centrally in the left eye and to I3 in the right eye. Overall, after rituximab, vision improved from 20/200 to 20/100 in the right and from 20/150 to 20/80 in the left eye; ERG responses also improved bilaterally and stabilized. This patient was thus determined as showing improvement on rituximab.

#### Patient 5 (P5)

**Fig. 7:**
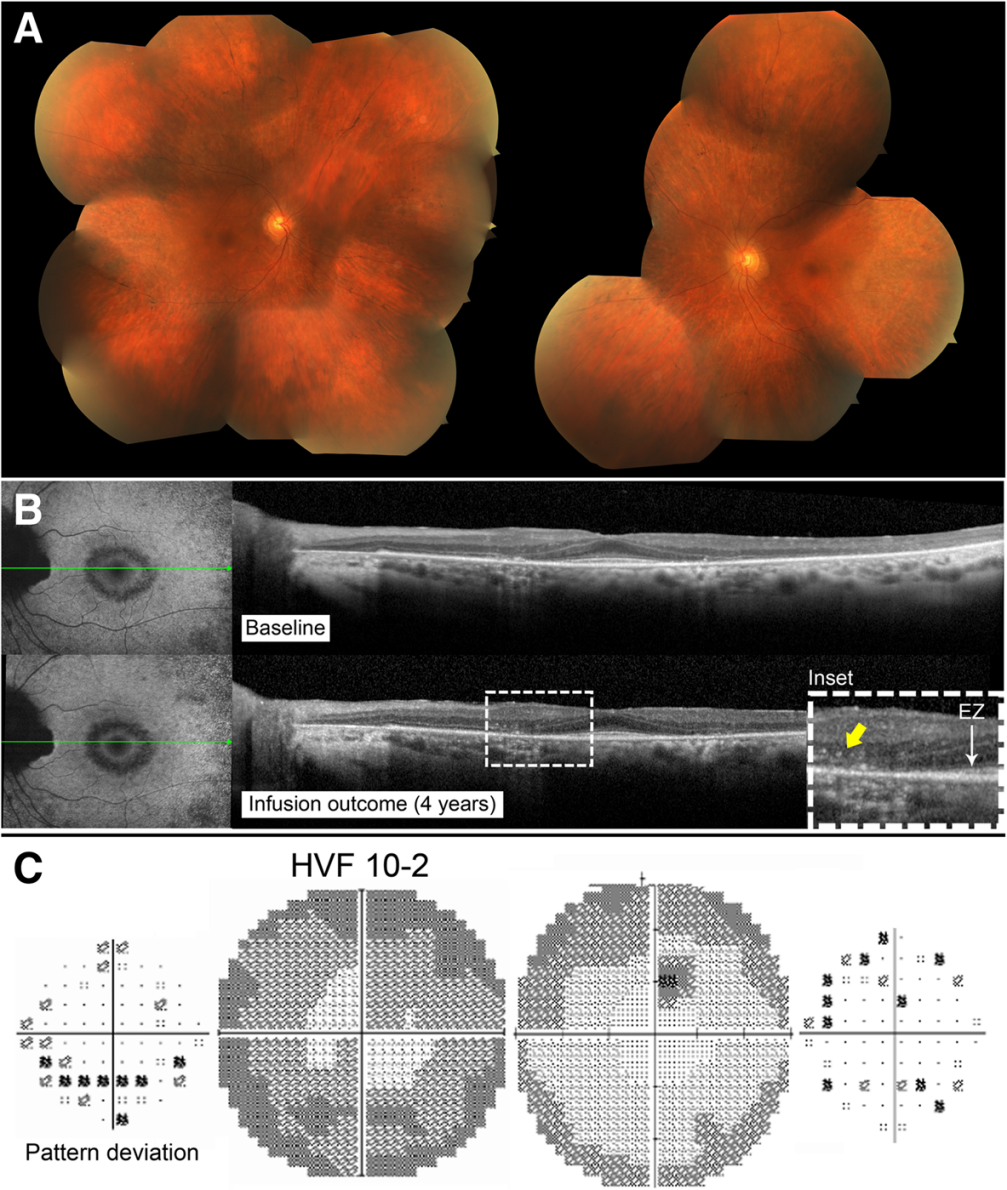
P5 imaging and functional assessments. P5 fundus pictures OU (**a**). OCT line OD showing stability over many years when comparing OCT at presentation (2011, *top*) and after rituximab initiation (2015, *bottom*) (**b**). Humphrey visual field 24–2 grey scale and pattern deviation OD before (2014, *left*) and after (2015, *right*) initiation of rituximab showing mild improvement on overall sensitivity (**c**)

A 70 year-old woman with known numerous autoimmune disorders reported night blindness for a few years, but blind spots in both eyes caused her to seek care. In December 2011, her vision was recorded at 20/25 in the right and 20/30 in the left eye. Funduscopic examination revealed extensive mottling of the RPE in the periphery, with limited peri-vascular pigment migration and vessel attenuation (Fig. 7a). ffERG showed an extinguished rod response, electronegative maximal response, and 30 Hz flicker response amplitudes that were around 12 μV bilaterally (Fig. 1e). Immunoblot analysis showed reactivity against 42-kDa (arrestin) proteins, and a neoplastic and infectious work-up was negative. Visual field worsened, which prompted the initiation of mycophenolate mofetil 500 mg twice daily eight months later. Rod-specific ERG responses improved initially, but eventually, visual field showed continued progression even with increased dosage of mycophenolate mofetil and the addition of oral prednisone. Repeated immunoblot analysis showed reactivity against multiple antigens, and repeated ERG showed continued deterioration in rod response. On September 2014, the patient’s immunosuppression treatment was replaced with three rituximab infusions administered over a period of three months. ERG responses fluctuated but remained stable. However, because of recurrent sinus infections, a second trial of rituximab was initiated only one year later after recurrence of visual symptoms. The patient subsequently developed ophthalmic zoster and nodular scleritis in the left eye. This prompted concern for an immunological deficiency secondary to rituximab, although her immunoglobulin levels were within the normal range, which was reassuring. Overall, the ERG, OCT (Fig. 7b), visual fields (Fig. 7c), and visual acuity in the right eye remained stable with rituximab, and the patient continued the medication. Visual acuity was slightly decreased in the left eye because of a cataract. This patient was thus classified as stabilized on rituximab.

## Discussion

AIR is a rare autoimmune disorder characterized by the production of ARAs that target retinal proteins. Normally when the body encounters a foreign pathogen, B cells bind to a unique antigen, which causes them to mature into antibody-producing plasma cells or memory B cells, which trigger an immune response. However, in autoimmune conditions such as AIR, the B cells become reactive to self-antigens [25] and begin to produce pathogenic ARAs. This process eventually induces retinal cell death and leads typically to a more rapid and progressive vision loss [2] compared to hereditary retinal degenerations, which show a slow mean decrease of 10% per year on 30 Hz flicker ERG response in patients with retinitis pigmentosa, for example [26].

In this case series, we exclusively studied patients with npAIR, which distinguishes itself from other forms of AIR by the lack of malignancy. In our cohort of npAIR patients, a high percentage (80%) were female, which is typical of autoimmune conditions [27], and 80% had another coexisting autoimmune condition, which is also reported in patients with AIR according to previous studies [27]. All patients had abnormal ERG responses, rapid disease progression and positive testing for ARAs. Four patients were previously taking an alternative immunosuppressant, and three initially responded well, although the beneficial effects eventually subsided. A recent study reported that in a subgroup of npAIR patients, roughly 63% responded well to immunosuppressive drugs such as cyclosporine, mycophenolate mofetil, infliximab, IVIg or steroids [27]. Although this estimate is higher than what was observed in other studies [6], patients’ variable and transient response to immunosuppression is not surprising given the incertitude in the pathophysiology of this disease, including the questionable pathogenicity of many subtypes of ARAs to the retina [28, 29]. Frequent failure of treatment has encouraged the pursuit of alternative drug strategies, rituximab being one example.

Rituximab is an immunosuppressant that has been used more recently in a number of systemic conditions, including patients with IgG4-related orbital diseases [30], myasthenia Gravis [31], neuromyelitis optica [32], and other ocular inflammatory or autoimmune conditions [33]. Binding of the drug to cell receptors leads to a rapid depletion in the population of B cells for approximately 6–12 months [34]. It is hypothesized that rituximab induces B-cell apoptosis through the activation of mitogen-activated protein kinases, natural killer cells, or the complement cascade [11].

In this case series, we found variable responses among five npAIR patients taking rituximab. Two appeared to stabilize, while one marginally improved and two others did not respond. Although treatment regimen varied between patients, all of them reached adequately low levels of B cells, confirming the potency of the drug. There are several possible reasons why treatment outcomes were inconsistent among patients. As mentioned, four patients had previously been prescribed immunosuppressants, and while three responded initially, they eventually became resistant to treatment. We hypothesize that improvement on rituximab may have been hampered by the limited number of functional photoreceptors or connected secondary-order cells remaining in the retinas of these patients. Additionally, each patient’s genetic and immunological backgrounds may confer greater or lesser amenability of immunotherapy (including to rituximab) for the treatment of their condition, as has been suggested for other immunologically based retinal diseases [35]. In fact, some studies explain the low sensitivity to rituximab by citing differences in B cell memory capacity for reconfiguration [36] or the lack of specific complement regulatory proteins on the cell’s surface [37] due to genetic differences among patients.

In addition, we did not observe a correlation between outcomes and changes in the type or level of antibodies after treatment in four out of four tested patients, which is unexpected. Indeed, in the two patients declared stable on rituximab, types (and level for one patient) of antibodies were similar at 5 and 8 months after starting rituximab. This might be because the CD20 receptor is not present on antibody-producing plasma cells, and thus, levels of immunoglobulin are not expected to decrease after rituximab infusion [34]. This phenomenon was observed in other studies as well. Looney et al. [38] reported improvement in patients with Lupus erythematosous following rituximab without changes in anti-double stranded DNA antibody or complement level. While Jarius et al. reported decreased antibody titers after rituximab treatment in neuromyelitis optica, the antibody always remained detectable in almost all patients [39]. Perhaps stability following treatment without change in antibody levels may be due to decreased antigen presentation rather than antibody level, [40] i.e. rituximab may be acting not only on B cell depletion, but on T-cell action as well [11]. Additionally, in P1, ARAs were still measurable after 7 months on rituximab but became undetectable 14 months later. Further studies are indicated to identify the appropriate time interval for repeat testing after baseline, although our data suggest that early on, antibody levels may not serve as a suitable proxy for patients’ response to rituximab. Instead, this test may be best interpreted in conjunction with other indices of retinal function and structure (BCVA, ffERG, multi-modal imaging, etc.).

ERG is a relatively objective test that not only serves as an important tool in diagnosing AIR, but also enables assessment of the severity of retinal dysfunction. Monitoring patients’ response to treatment is challenging, as many visual tests such as BCVA and visual field exams are subjective and vary based on the patient’s affect, learning curve for complex tests, and cooperation [41–43]. For example, Mizener et al. observed that ERG was more sensitive than visual fields in assessing progression of three patients with npAIR [2]. Despite some variability between sessions, ERG testing in this study also proved to be an effective tool for monitoring patients over time, and changes in ERG outcomes correlated closely with patient symptoms [44], thus highlighting the utility of this test.

ERG data on npAIR patients is not as abundant as it is for patients with CAR [45–47] and MAR [48]. In general, while some patients present with greater rod than cone dysfunction initially, for a small minority, cones are affected first [8]. In this case series, four out of five patients presented with moderate to severe rod-cone dysfunction on ffERG, while only one showed cone-rod dysfunction initially. An electronegative appearance on the maximal response was detected in P1 and P3, suggesting that the inner retinal layers were most affected. The variable effect of different ARAs in each patient may explain this disturbance pattern wherein the inner retinal layers are targeted, which has been demonstrated frequently in MAR but is less commonly reported in CAR [49] and npAIR [2]. With progression of the disease, the ERG recording eventually becomes extinguished in most patients. Our findings demonstrate that ERG is an effective strategy for monitoring npAIR patients over time in an objective manner that facilitates clinical decision making by supplementing findings from retinal imaging, BCVA, and visual field tests.

Thus far, two case reports have been published studying the response of patients with npAIR to rituximab, and in each [14, 16], patients were reported to benefit from the drug, with overall improvements in retinal function. Additionally, one case series studied six npAIR patients receiving rituximab and/or combination therapy [19]. They found that following mono- or combinatorial therapy, 66.7% of eyes had stable visual acuity, 50% showed stability on visual field testing, and 33.3% showed stability or improvement on ERG. They also found that at least one pathogenically proven ARA band resolved after treatment. Overall, they concluded that stability or improvement on two or more tests in 83.5% of patients could be considered a successful treatment. Contrastingly, we observed much more variability among patients in our cohort and concluded that only 60% were stabilized or improved following treatment.

There are several notable differences in study design that may account for the differences observed in our findings. The primary difference lies in the standardization of the dosage of rituximab, which was administered at 375 mg/m^2^ every week for 8 weeks, then 375 mg/m^2^ monthly [50, 51]. Contrastingly, patients in our study were treated with rituximab in a customized fashion based on symptoms and specialist preferences. A secondary difference is their combinatorial approach, wherein rituximab was co-administered with oral cyclophosphamide or bortezomib in 4 out of 6 of their patients, while ours were treated with rituximab exclusively. However, there are many points of consensus between our studies: visual acuity was on average stabilized in both cohorts, adverse events occurred in a minority of patients, and we both found unpredictable results in ARA outcomes, making their interpretation challenging. Additionally, our findings build on Foster et al.’s by supplying OCT and ARA titer testing results, although repeated testing among larger cohorts is still greatly needed.

Some limitations of our study should be acknowledged. While one of our patients was under 10 years of age and one older than sixty, the typical npAIR diagnosis is made between the ages of 20 and 25 years of age. Additionally, the regiment of rituximab was not standardized across patients. Some patients followed the rituximab protocol that was developed for B-cell lymphoma, while some were prescribed a regiment that was originally designed for rheumatoid arthritis patients, and yet others used non-standard protocols. Additional experience with other patients with autoimmune disorders and retinal atrophies may guide dosing of rituximab in the future. Additionally, the time point of assessment and antibody testing following rituximab infusions were also different for each patient, and the optimal follow-up time after infusion cannot be determined from these data. Testing blood before and at varying intervals after each rituximab infusion is one strategy that could determine the ideal timing for ARA testing in future studies, which in turn may enable better delineation of the drug’s effects on ARAs. Finally, ratio analysis for ERG was used in this series in order to obtain an efficient comparison strategy of responses at different points for each patient, although actual voltage numbers could also have been used.

## Conclusions

Overall, our findings suggest that rituximab may stabilize the progression of retinal dysfunction in some patients with advanced npAIR, although expectations for improvement should be temperate. The recommended interval for ARA testing following rituximab administration, as well as the use of this measure to drive decision making in isolation from other tests, remains an important consideration for future studies. To obtain an approximation of patients’ progression and response to treatment, ffERG 30 Hz-flicker can be used alongside other assessments, like ARA measures, to obtain a complete overview of patients’ progression and response to treatment. Several patients initially responded well to other immunosuppressive therapies, suggesting that treatment should be readily considered rather than mere observation of the patient. In future studies, it would be valuable to explore whether earlier administration after diagnosis may enable greater improvements in patient outcomes.

## Abbreviations

AIR: Autoimmune retinopathy
ARAs: Antiretinal antibodies
B-cells: B lymphocytes
BCVA: Best corrected visual acuity
CAR: Cancer-associated retinopathy
ERG: Electroretinogram
EZ: Ellipsoid zone
ffERGs: Full-field electroretinograms
HVF: Humphrey visual field
ISCEV: International Society for Clinical Electrophysiology of Vision
IVIg: Intravenous immunoglobulins
MAR: Melanoma-associated retinopathy
NCL: Neuronal ceroid lipofuscinose
npAIR: Non-paraneoplastic autoimmune retinopathy
NYPH: New York Presbyterian Hospital
OD: Right eye
OS: Left eye
pAIR: Paraneoplastic autoimmune retinopathy
RPE: Retinal pigment epithelium
SD-OCT: Spectral-domain optical coherence tomography
μV: Microvolts
